# Childhood Obesity and Breastfeeding Rates in Pennsylvania Counties—Spatial Analysis of the Lactation Support Landscape

**DOI:** 10.3389/fpubh.2020.00123

**Published:** 2020-04-21

**Authors:** Anna Blair, Ellie MacGregor, Nikki Lee

**Affiliations:** ^1^Healthy Children Project, East Sandwich, MA, United States; ^2^Academy of Lactation Policy and Practice, Forestdale, MA, United States; ^3^Private Practice Lactation Consultant, Elkins Park, PA, United States

**Keywords:** breastfeeding, childhood obesity, lactation support provider, certified lactation counselor, international board certified lactation consultant

## Abstract

Childhood obesity is a global public health issue. As the prevalence of childhood obesity continues to rise, identification of potential interventions by public health policy makers, and health care providers is imperative. Breastfeeding, the most optimal method of infant feeding, has been demonstrated to protect against childhood obesity. Lactation support providers (LSPs) play a key role in providing education, care, and support to families considering a feeding choice. Access to professional lactation care increases breastfeeding initiation, exclusivity, and duration rates, regardless of the credential that the LSP holds. The aims of the current study were to examine the relationship between childhood obesity and breastfeeding rates in Pennsylvania (PA) counties; to examine the relationship between geographic access to professional LSPs in PA counties and breastfeeding rates; and to examine the relationship between geographic access to professional LSPs and childhood obesity in PA counties. Data were collected on 617 professional LSPs in 67 PA counties. There are 608 Certified Lactation Counselors (CLCs) and 144 International Board Certified Lactation Consultants (IBCLCs) in PA. County-level breastfeeding rates, childhood obesity rates, and the number of CLCs and IBCLCs were tested for significance at the *p* < 0.01 level using a two-tailed significance test and bivariate Pearson's correlation. The results show a significant, inverse relationship between breastfeeding rates and childhood obesity prevalence at the county level, *p* < 0.01. There is also a significant, inverse relationship between the number of CLCs and the number of all professional LSPs and childhood obesity rates at the county level, *p* < 0.01. Thus, the availability of breastfeeding support is significantly related to breastfeeding rates and inversely related to childhood obesity rates across Pennsylvania.

## Introduction

Obesity is a global public health crisis and is strongly correlated with chronic health conditions such as diabetes, cardiovascular disease, and high blood pressure. Among children and adolescents aged 2–19 years, obesity rates are on the rise (1). According to the World Health Organization (WHO), 41 million infants and young children were overweight or obese in 2016 (2). There has been a ten-fold increase in the number of obese children and adolescents over the last four decades (3). The Centers for Disease Control and Prevention (CDC) National Center for Health Statistics (NCHS) found the obesity prevalence was 13.9% among 2- to 5-year-olds, 18.4% among 6- to 11-year-olds, and 20.6% among 12- to 19-year olds. Hispanic youth (25.8%) and non-Hispanic African American youth (22.0%) had a higher prevalence of obesity than among non-Hispanic white (14.1%) youth and non-Hispanic Asian (11.0%) youth (4).

Interventions to reduce childhood obesity are multi-faceted and must include several components. Among these interventions, breastfeeding—a behavioral variable—has been shown to decrease the risk of childhood obesity and is inversely associated with weight gain velocity and BMI among infants (5–7). While any breastfeeding is protective against childhood obesity, the WHO recommends exclusive breastfeeding for 6 months, as it conveys the strongest protective effect (8). In a recent meta-analysis, recent review, researchers concluded that exclusively breastfed infants have a 31% lower chance of later developing overweight and obesity (9). In addition, formula-fed infants consume more milk and gain weight more rapidly than breastfed infants (10). The National Academy of Medicine recommends breastfeeding as part of a broader strategy to preventing childhood obesity and recognizes that the U.S. is falling short in its efforts to support breastfeeding (11). Breastfeeding is part of the CDC's plan for state implementation of obesity prevention strategies which include nutrition, breastfeeding, physical activity, and decreased screen time (12). The promotion, protection and support of breastfeeding for the first 6 months is a critically important public health priority.

Despite the evidence that breastfeeding is the optimal feeding method for infants, breastfeeding rates remain low across the U.S. According to the CDC's 2018 Breastfeeding Report Card, among infants born in 2015 in the United States, 4 out of 5 (83.2%) started to breastfeed, but just over half (57.6%) were breastfeeding at 6 months, and only about one-third (35.9%) continued breastfeeding to 12 months (13). When parents receive support from a professional LSP who provides education and counseling during the breastfeeding process, breastfeeding initiation and duration rates increase (14). Interventions provided by LSPs within their respective Scopes of Practice, such as implementing the best practice of placing mother and baby skin-to-skin for the first hour immediately following birth, educating a family about early infant feeding cues, and delivering face-to-face anticipatory guidance to families have all been shown to increase breastfeeding duration and exclusivity (15, 16).

The United States Breastfeeding Committee (USBC), the American College of Obstetricians and Gynecologists (ACOG), and the CDC recognize Certified Lactation Counselors (CLCs) and International Board Certified Lactation Consultants (IBCLCs) as professional breastfeeding supporters (17–19). However, there are currently only 4.57 CLCs and 3.79 IBCLCs per 1,000 live births in the U.S. (20).

Informed by this evidence, the aims of the current study were: to examine the relationship between childhood obesity and breastfeeding rates in PA counties; to examine the relationship between geographic access to professional LSPs in PA counties and breastfeeding rates; and to examine the relationship between geographic access to professional LSPs and childhood obesity in PA counties.

## Materials and Methods

### Design

This is a cross-sectional, observational study using retrospective data.

### Setting

PA is estimated to have a population of 12.81 million according to projections from the 2010 U.S. Census Bureau data. This makes PA the sixth most populous state in the country. 8.9 million residents live in urban areas of the state and 2.7 million residents live in the rural areas of the state. As of July 1, 2018, 81.8% of the population in PA identified as White, 12% identified as Black or African American, and 7.6% identified as Hispanic or Latino (21).

According to the PA Department of Health, 16.8% of children in grades K-6 during the 2017–2018 school year were considered obese (22). Among children born in 2015, 83.8% breastfed at least once, 59.2% were breastfeeding at 6 months and 39% were breastfeeding at 12 months (23).

According to the most recent publicly available data, there are 608 CLCs and 144 IBCLCs in PA. With 137,682 live births in 2017, this indicates that there are 4.42 CLCs per 1,000 births and 1.05 IBCLCs per 1,000 births in the state (24–27).

### Measurement

We measured the distribution of CLCs and IBCLCs, county level breastfeeding rates, and county level childhood obesity rates across PA. Data were collected from multiple sources. The obesity rates of children in grades K-6 were published by the Pennsylvania Department of Health in September 2019. The percent of live births to mothers who breastfed in PA was published in February 2018 by the PA Department of Health (28). The total births by county in Pennsylvania were published in 2018 by the PA Department of Health (29).

The number of CLCs in PA was obtained on December 2, 2019 from the Academy of Lactation Policy and Practice (ALPP) (30). CLCs were assumed to have remained in the zip code listed when they obtained their certification. The number of IBCLCs was obtained from the International Lactation Consultant Association (ILCA) and combined with data obtained from the United States Lactation Consultant Association (USLCA) on December 2, 2019 (31). To find the number of IBCLCs on ILCA, a 283-mile search radius was used around Philadelphia, Pittsburg, and Beaver Springs. These names were cross-referenced with one another to ensure that each individual was counted only once. To find the number of IBCLCs on USLCA, a 10-mile search radius was used to search each unique zip code within the state. These names were cross-referenced with one another to ensure that each individual was counted only once. Next, the IBCLCs populated from both directories were cross-referenced to ensure that each individual was counted only once. Each individual was then counted as a LSP in their respective county. The density percentage of CLCs and IBCLCs was calculated by county to demonstrate the prevalence of professional LSPs per live births. This density percentage was calculated by taking the number of LSPs per county and dividing the total live births per county. Data on the definitions of rural, urban, and suburban counties in Pennsylvania were obtained from The Center for Rural Pennsylvania (32). Counties were designated as urban or rural in Table 1.

**Table 1:** Professional lactation support providers in PA counties.

**County**	**Designation**	**Live births (N)**	**Breast-feeding initiation (%)**	**Childhood obesity (%)**	**CLC^**a**^ (N) (Density %)**	**IBCLC^**b**^ (N) (Density %)**	**Total LSP^**c**^ (N) (Density %)**
Adams	Rural	934	85.3	18.4	6 (0.6%)	3 (0.3%)	9 (1.0%)
Allegheny	Urban	12,872	79.9	14.2	67 (0.5%)	19 (0.1%)	86 (0.7%)
Armstrong	Rural	560	64.8	18.4	1 (0.2%)	0 (0%)	1 (0.2%)
Beaver	Urban	1,584	73.4	19.5	3 (0.2%)	1 (0.1%)	4 (0.3%)
Bedford	Rural	468	74.8	21.6	4 (0.9%)	0 (0%)	4 (0.9%)
Berks	Urban	4,608	77.8	19.4	9 (0.2%)	5 (0.1%)	14 (0.3%)
Blair	Rural	1,210	76.7	19.5	17 (1.3%)	2 (0.2%)	19 (1.6%)
Bradford	Rural	672	76.9	19.6	5 (0.7%)	0 (0%)	5 (0.7%)
Bucks	Urban	5,712	82.8	13.8	16 (0.3%)	15 (0.3%)	31 (0.5%)
Butler	Rural	1,784	81.8	15.0	6 (0.3%)	0 (0%)	6 (0.3%)
Cambria	Rural	1,209	74.5	18.3	16 (1.3%)	0 (0%)	16 (1.3%)
Cameron	Rural	37	61.1	21.5	1 (2.7%)	0 (0%)	1 (2.7%)
Carbon	Rural	547	72.9	19.4	1 (0.2%)	0 (0%)	1 (0.2%)
Center	Rural	1.173	88.9	12.9	6 (0.5%)	6 (0.5%)	12 (1.0%)
Chester	Urban	5,378	91.4	11.2	27 (0.5%)	5 (0.1%)	32 (0.6%)
Clarion	Rural	370	68.9	20.1	1 (0.3%)	0 (0%)	1 (0.3%)
Clearfield	Rural	700	72.7	22.3	5 (0.7%)	0 (0%)	5 (0.7%)
Clinton	Rural	419	84.4	22.3	0 (0%)	0 (0%)	0 (0%)
Columbia	Rural	522	80.3	20.8	2 (0.4%)	0 (0%)	2 (0.4%)
Crawford	Rural	913	81.0	21.3	1 (0.1%)	0 (0%)	1 (0.1%)
Cumberland	Urban	2,598	88.5	14.7	13 (0.5%)	4 (0.2%)	17 (0.7%)
Dauphin	Urban	3,402	85.0	17.9	20 (0.6%)	3 (0.1%)	23 (0.7%)
Delaware	Urban	6,470	84.5	14.5	33 (0.5%)	11 (0.2%)	44 (0.7%)
Elk	Rural	263	61.5	16.8	4 (1.5%)	0 (0%)	4 (1.5%)
Erie	Urban	2,785	67.2	18.4	17 (0.6%)	2 (0.1%)	19 (0.7%)
Fayette	Rural	1,301	61.2	21.1	5 (0.4%)	1 (0.1%)	6 (0.5%)
Forest	Rural	19	76.0	24.2	0 (0%)	0 (0%)	0 (0%)
Franklin	Rural	1,728	89.7	18.8	6 (0.3%)	0 (0%)	6 (0.3%)
Fulton	Rural	141	78.9	20.3	1 (0.7%)	0 (0%)	1 (0.7%)
Greene	Rural	335	54.4	20.9	1 (0.3%)	0 (0%)	1 (0.3%)
Huntingdon	Rural	417	71.7	18.2	3 (0.7%)	0 (0%)	3 (0.7%)
Indiana	Rural	809	80.6	19.5	2 (0.2%)	1 (0.1%)	3 (0.4%)
Jefferson	Rural	477	77.4	22.1	3 (0.6%)	0 (0%)	3 (0.6%)
Juniata	Rural	293	84.8	23.1	1 (0.6%)	0 (0%)	1 (0.6%)
Lackawanna	Urban	2,027	67.4	21.1	7 (0.3%)	0 (0%)	7 (0.3%)
Lancaster	Urban	6,954	87.6	15.3	32 (0.5%)	6 (0.1%)	38 (0.5%)
Lawrence	Rural	879	69.4	21.2	2 (0.2%)	0 (0%)	2 (0.2%)
Lebanon	Urban	1,555	85.1	20.0	5 (0.3%)	4 (0.3%)	9 (0.6%)
Lehigh	Urban	4,375	83.9	17.1	28 (0.6%)	4 (0.1%)	32 (0.7%)
Luzerne	Urban	3,317	68.9	19.4	6 (0.2%)	0 (0%)	6 (0.2%)
Lycoming	Rural	1,210	84.3	19.9	9 (0.7%)	1 (0.1%)	10 (0.8%)
McKean	Rural	365	68.5	21.3	5 (1.4%)	0 (0%)	5 (1.4%)
Mercer	Rural	1,062	72.7	19.5	0 (0%)	1 (0.1%)	1 (0.1%)
Mifflin	Rural	610	82.2	19.1	2 (0.3%)	0 (0%)	2 (0.3%)
Monroe	Rural	1,521	82.2	21.3	3 (0.2%)	0 (0%)	3 (0.2%)
Montgomery	Urban	8,538	91.2	13.6	37 (0.4%)	23 (0.3%)	60 (0.7%)
Montour	Rural	197	91.1	17.4	3 (1.5%)	0 (0%)	3 (1.5%)
Northampton	Urban	2,885	83.4	18.5	11 (0.4%)	0 (0%)	11 (0.4%)
Northumber-land	Rural	895	78.1	21.0	1 (0.1%)	1 (0.1%)	2 (0.2%)
Perry	Rural	506	80.6	17.7	1 (0.2%)	1 (0.2%)	2 (0.4%)
Philadelphia	Urban	20,654	80.9	17.4	79 (0.4%)	11 (0.1%)	99 (0.5%)
Pike	Rural	413	71.3	17.4	0 (0%)	0 (0%)	0 (0%)
Potter	Rural	168	76.0	20.8	0 (0%)	0 (0%)	0 (0%)
Schuylkill	Rural	1,275	68.6	21.1	4 (0.3%)	0 (0%)	4 (0.3%)
Snyder	Rural	432	84.7	20.2	0 (0%)	0 (0%)	0 (0%)
Somerset	Rural	738	75.9	21.5	1 (0.1%)	0 (0%)	1 (0.1%)
Sullivan	Rural	42	93.5	26.9	0 (0%)	0 (0%)	0 (0%)
Susquehanna	Rural	388	75.4	20.4	1 (0.3%)	0 (0%)	1 (0.3%)
Tioga	Rural	394	76.8	20.2	3 (0.8%)	0 (0%)	3 (0.8%)
Union	Rural	401	90.5	15.9	2 (0.5%)	0 (0%)	2 (0.5%)
Venango	Rural	471	67.4	20.7	0 (0%)	0 (0%)	0 (0%)
Warren	Rural	417	80.6	20.7	3 (0.7%)	1 (0.2%)	4 (1.0%)
Washington	Rural	2,017	72.6	15.6	13 (0.6%)	2 (0.1%)	15 (0.7%)
Wayne	Rural	396	73.1	18.7	4 (1.0%)	0 (0%)	4 (1.0%)
Westmoreland	Urban	2,914	74.9	17.6	5 (0.2%)	4 (0.1%)	9 (0.3%)
Wyoming	Rural	240	74.6	19.3	2 (0.8%)	0 (0%)	2 (0.8%)
York	Urban	4,711	85.6	16.3	45 (1.0%)	7 (0.1%)	52 (1.1%)
TOTAL	-	135,677	-	-	617	144	761

a *Certified lactation counselor*.

b *International board certified lactation consultant*.

c *Lactation support providers*.

### Data Analysis

To understand the relationship between childhood obesity and breastfeeding in PA counties, we ran a two-tailed significance test at the 0.01 level and a bivariate Pearson's correlation. We graphed the linear relationship on a scatter plot. To understand the relationship between geographic access to CLCs and childhood obesity by PA county, we ran a two-tailed significance test at the 0.01 level and a bivariate Pearson's correlation. We graphed the linear relationship on a scatter plot. Further, we examined the relationship between geographic access to CLCs and breastfeeding rates by PA county by running ANOVA tests to test for significant differences in the number of CLCs by PA county and the percentage of births to mothers who breastfed at the 0.05 level. This analysis was repeated to examine the relationship between all professional LSPs and breastfeeding rates by PA county. Results were mapped to demonstrate geographic access, or lack thereof, to LSPs across the state. To understand the relationship between professional lactation support and childhood obesity by PA county, we ran a two-tailed significance test at the 0.01 level and a bivariate Pearson's correlation. We graphed the linear relationship on a scatter plot. All data analysis was conducted using IBM SPSS Statistics version 26.0 (33).

## Results

**Figure 1:**
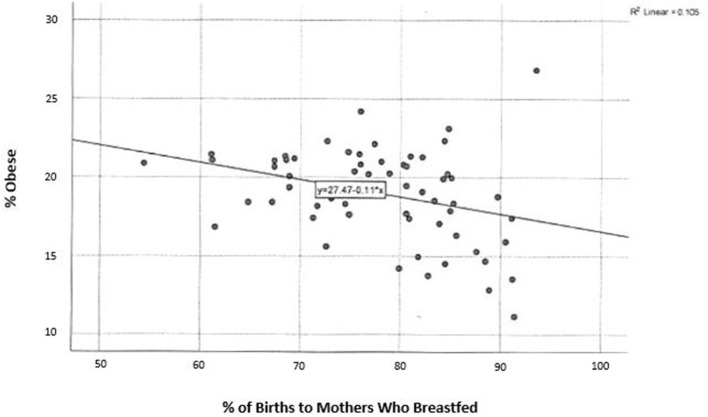
Percent of live births to mothers who breastfed and childhood obesity in Pennsylvania.

Among children in grades K-6, there is a significant, inverse correlation between childhood obesity and breastfeeding rates by county in PA, *r*_(66)_ = −0.395, *p* < 0.01 (Figure 1). During the 2017–2018 school year, 160,220 (16.8%) of children fell within, or above, the 95th percentile of weight (defined as obese). Rates of obesity were highest in the northwest area of the state.

**Figure 2:**
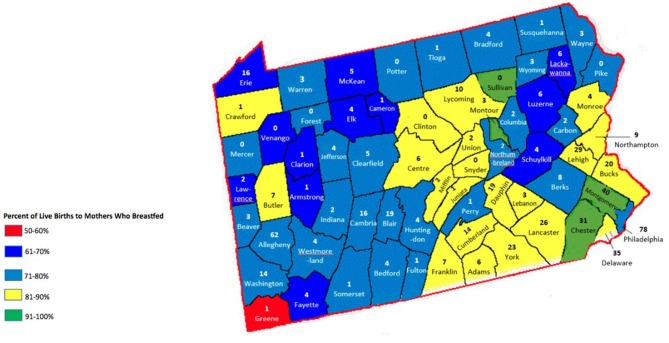
Certified lactation counselors in Pennsylvania counties.

LSPs are unequally distributed throughout the state, with more LSPs populated in urban areas. The least amount of CLCs are located in the northwest area, excluding Erie county (Figure 2). There is a significant, inverse correlation between obesity rates and geographic access to CLCs in the state, *r*_(66)_ = −0.526, *p* < 0.01 (Figure 4). According to the PA Department of Health, the southeastern counties of PA have the highest breastfeeding rates in the state (Table 1). In these counties, childhood obesity rates are the lowest among all geographic districts (15.2%).

**Figure 3:**
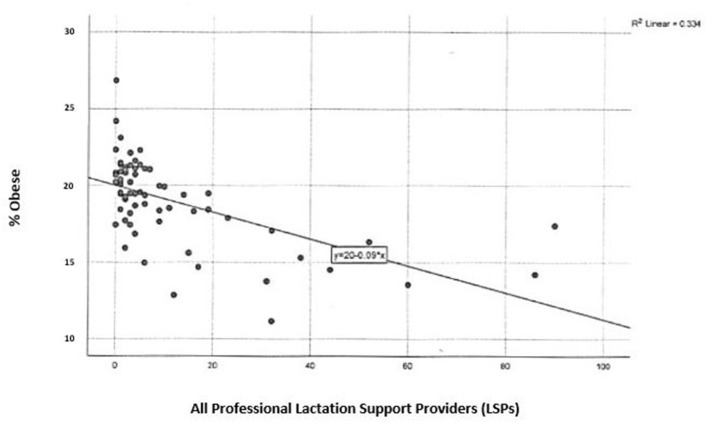
Professional lactation support providers and childhood obesity in Pennsylvania.

**Figure 4:**
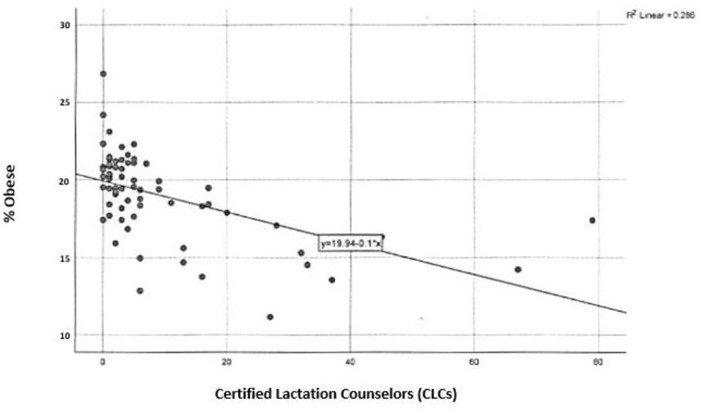
Certified lactation counselors and childhood obesity in Pennsylvania.

**Figure 5:**
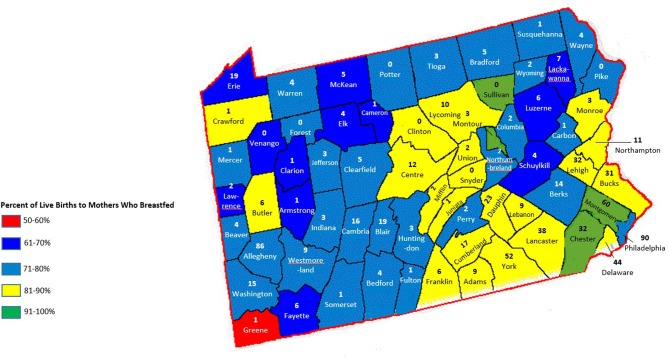
All lactation support professionals in Pennsylvania counties.

In counties where there are more CLCs and therefore families have greater geographic access to a CLC by proximity, breastfeeding rates are higher, *r*_(66)_ = 0.277, *p* < 0.05 (Figure 2). In counties where there are more professional LSPs, breastfeeding rates are the highest, *r*_(66)_ = 0.307, *p* < 0.05 (Figure 5).

There are 761 professional LSPs in PA, of which 617 are CLCs and 144 are IBCLCs (Table 1). The highest concentrations of all LSPs in PA are in the southeast district of the state and there are more CLCs spread throughout all counties in PA as compared to IBCLCs (Figure 5). There is a significant, inverse relationship between childhood obesity rates and geographic access to all professional LSPs in the state, *r*_(66)_ = −0.567, *p* < 0.01 (Figure 3).

## Discussion

The data we have examined show an association between low rates of childhood obesity in counties with more LSPs in PA. LSPs, such as CLCs and IBCLCs, who support families to achieve their personal breastfeeding goals, play a pivotal role in increasing breastfeeding initiation and continuation rates.

Childhood obesity is a complex public health issue. No single factor has led to the drastic increases in childhood obesity in the U.S. Rather, research suggests that complementary changes have simultaneously decreased a child's daily energy expenditure while increasing caloric intake (34). One's diet and level of physical activity—the two risk factors most strongly correlated to obesity—have changed significantly over the past two decades. While lifestyle is a primary factor contributing to obesity among adults, infant feeding choice is a primary factor contributing to childhood obesity in early life (35). The relationship between childhood obesity and breastfeeding rates observed in this study supports the existing evidence that breastfeeding and breastfeeding support by professional LSPs can be assumed to be a protective factor against childhood obesity across PA.

Breastfeeding as an infant feeding choice should be viewed as a public health strategy to address the rising rates of childhood obesity in PA. Equitable geographic access to professional lactation support, as examined in this study, is only one component contributing to the ability to seek care when breastfeeding. Paying for LSP care may also affect a family's decision to seek help and support (36). In rural PA counties, the average household income for families is generally lower than that of families in urban counties across the state (37). Financial accessibility may be a primary consideration when accessing lactation support in rural communities.

Passed in 2010, the Affordable Care Act (ACA) requires insurers to provide coverage of breastfeeding supplies and support services (38). As some post-implementation analyses have shown, breastfeeding initiation rates did increase post-ACA implementation (39). Unfortunately, many private and public insurance companies are not paying for lactation care and services and have not reimbursed their policyholders for these costs if incurred. With limited geographic access to a professional LSP and the financial strain of receiving support, families in rural counties in PA may be discouraged from seeking help and, as a result, may not continue to breastfeed their children. As demonstrated in the current study, rural counties in PA, specifically those in the northwest district, have the highest rates of childhood obesity across the state and the lowest density of professional LSPs.

Structural barriers, such as a lack of Baby-Friendly designated hospitals, may also have an effect on breastfeeding initiation and continuation rates (40, 41). The Baby-Friendly Hospital Initiative was launched to establish environments and educational services that enhance breastfeeding experiences for new parents. The overarching goal of the initiative is to improve breastfeeding outcomes, including increased initiation, duration, and exclusivity rates. There are 14 (5.6%) Baby-Friendly designated hospitals in Pennsylvania out of the total 90 hospitals/birthing centers. Comparatively, 16.7% of U.S. hospitals/birthing centers are designated Baby-Friendly (42). In addition, 20 hospitals have received Keystone 10 designation, of which 14 are also Baby-Friendly designated. The Keystone 10 Initiative is a quality improvement initiative aimed at supporting breastfeeding families in PA through improvements to birthing facilities (43). Eighty-four out of the 90 hospitals/birthing facilities have started the process for improving maternity care services that support breastfeeding by enrolling in the Keystone 10 program. To date, less than half have completed 6 of the 10 steps to fulfill the requirements for Keystone designation. Of the 14 designated hospitals in PA, only one is located in a rural county (44). One remedy to address this disparity might be policies directed at increasing the number of Baby-Friendly hospitals through incentivizing the designation process for hospitals in rural counties (45).

### Limitations

Several limitations to this study exist. First, the current study only examines geographic access to CLCs and IBCLCs and breastfeeding rates in PA. There is no data included to analyze access to La Leche League Leaders, WIC peer counselors, breastfeeding educators, doulas, lactation educators, or any other LSP in the community. The influence of breastfeeding support of any kind on breastfeeding rates should not be understated.

The IBCLC data were collected using publicly available data from the ILCA and USLCA online search databases, which are membership organizations. If an IBCLC is not listed as a provider in their county on the ILCA or the USLCA websites, they were not included in the total number of IBCLCs per county. A preferred data source using per county counts from the International Board Certified Lactation Consultant Examiners (IBLCE) was not made available for this research.

The current study utilizes retrospective data to assess the relationship between childhood obesity prevalence, breastfeeding rates, and geographic access to professional lactation support. Due to the nature of the available data, potential confounding variables were not controlled for. The strength of the current study and its results validate the need for a future, prospective study on this subject where potential confounding variables can be controlled.

Finally, many LSPs may hold their LSP credential, but may not be actively working in that role. These individuals were counted as a LSP in PA, but may be working in a different role, with limited interaction between themselves and breastfeeding families.

The facilitation of breastfeeding and assisting families in reaching their breastfeeding goals often requires support beyond the immediate postnatal period. Lactation support, counseling, and education provided by a CLC or an IBCLC is just one of many factors contributing to a successful breastfeeding experience.

## Conclusions

We examined the relationship between breastfeeding rates, childhood obesity, and breastfeeding support from a CLC and from all professional LSPs in PA counties. Clearly, available breastfeeding support is significantly related to an increase in breastfeeding rates and a decrease in childhood obesity rates across PA. This analysis can be replicated in other states to increase equitable access to lactation support and counties in PA can use this blueprint as a strategy to increase breastfeeding rates and decrease rates of childhood obesity.

## Data Availability Statement

All datasets generated for this study are included in the article/supplementary material.

## Ethics Statement

Ethical approval for this study was not required in accordance with local legislation and national guidelines. No human studies are presented in this manuscript. This retrospective study involved the counts of individuals in the public arena using only publicly available or accessible records without contact with the individual/s. The current research is based on a review of published/publicly reported literature.

## Author Contributions

AB and EM: conceptualization. AB, EM, and NL: data curation, methodology, and writing—review and editing. EM: formal analysis and software and writing—original draft.

## Conflict of Interest

The authors declare the following areas of interest: the authors hold the professional LSP credentials, CLC, and IBCLC. One author was employed by the Academy of Lactation Policy and Practice (issuer of the credential CLC), one author was employed by the Healthy Children Project, Inc. (an organization that provides training for both the CLC and IBCLC examination preparation), and one author holds the credential ANLC and is a lactation consultant in private practice in PA.
